# Interprofessional Perceptions and Collaboration Between Medicine and Dentistry in Croatia: A Qualitative Study of Faculty and Student Perspectives

**DOI:** 10.3390/healthcare14142097

**Published:** 2026-07-14

**Authors:** Zora Tomić, Anita Lauri Korajlija, Ivana Šutej

**Affiliations:** 1 Health Centre of the Croatian, Department of Internal Affairs, 10000 Zagreb, Croatia; 2 Department of Psychology, Faculty of Humanities and Social Sciences, University of Zagreb, 10000 Zagreb, Croatia; 3 Department of Pharmacology, School of Dental Medicine, University of Zagreb, 10000 Zagreb, Croatia

**Keywords:** interprofessional relations, medical education, dental education, collaboration, qualitative research, Croatia

## Abstract

**Background/Objectives:** Collaboration between medical and dental professionals is essential for comprehensive, patient-centred healthcare, yet the two fields often remain separate in education and practice. This study explored attitudes, experiences, and perceptions of interprofessional relationships among medical and dental faculty and students at a Croatian university. **Methods:** A qualitative study using semi-structured interviews was conducted. Thirty-nine participants, including faculty members and students from medicine and dentistry, were interviewed. Data were analyzed using thematic analysis to identify patterns and themes related to interprofessional perceptions and collaboration. **Results:** Participants generally reported mutual respect, although their accounts revealed subtle hierarchies, persistent stereotypes, and implicit biases. Overt animosity was rare, often limited to humour, though instances of disrespect and status differentiation were described. Educational collaboration was minimal, with siloed curricula and few structured initiatives, while clinical collaboration was described as largely case-dependent and centred on referral rather than coordinated care. Personal exposure to the other profession mitigated some biases, suggesting that social proximity influences professional perceptions. **Conclusions:** Despite overall respect, participants identified structural, cultural, and educational factors that limited opportunities for interprofessional engagement within the studied setting. The findings suggest that creating opportunities for structured interprofessional learning and collaborative clinical practice may strengthen professional understanding, support more integrated models of care, and ultimately contribute to improved patient-centred healthcare.

## 1. Introduction

Interprofessional collaboration (IPC) is widely recognized as essential for safe, high-quality, and patient-centred healthcare delivery. There is a growing international consensus that interprofessional education (IPE) during undergraduate training is vital for fostering collaboration and reducing barriers between healthcare providers [1]. While substantial attention has focused on collaboration between medicine, nursing, and allied health professions, relatively little research has examined the interface between medicine and dental medicine, despite well-established links between oral and systemic health [2,3]. Furthermore, most existing studies have focused on quantitative assessments of interprofessional education or attitudes, whereas qualitative research exploring how medical and dental students and faculty members construct professional identities, boundaries, and collaborative relationships remains limited.

Although both medical doctors and doctors of dental medicine undergo rigorous scientific education and hold doctoral degrees, they function as distinct regulated health professions in many countries, including Croatia. In the Croatian context, medicine and dental medicine are delivered through separate faculties with largely non-overlapping curricula, which results in minimal shared educational exposure. Medical students receive extensive training in systemic diseases but limited structured education in oral health, while dental students focus intensively on oral and maxillofacial conditions with comparatively limited integration into broader medical training [4]. Such structural separation may contribute to fragmented care and reduced understanding of each profession’s scope of practice [2,3].

Beyond structural division, professional relationships are shaped by social and cultural processes. Research indicates that stereotypes regarding curricular difficulty, professional prestige, and scope of practice may develop early during training [5]. Medical students may perceive dentistry as a narrower field focused primarily on teeth, while viewing their own education as broader or more demanding; conversely, dental students may experience their expertise as undervalued or dismissed. A recent review highlighted that misunderstandings regarding professional roles, combined with limited intergroup contact, can contribute to intergroup bias, stress, and even bullying behaviours within medical–dental student interactions [6]. These perceptions are not merely individual attitudes, but may reflect broader social identity processes in which group membership shapes in-group favouritism and out-group differentiation.

Empirical evidence from other contexts supports this interpretation. For example, a national survey in Nigeria found that interprofessional conflict and rivalry were widely perceived among healthcare professionals, with many participants reporting that these tensions negatively affected clinical practice and patient care. The study also highlighted the influence of professional hierarchies and organizational structures on interprofessional relationships [7]. Similar patterns of competitiveness and perceived hierarchy have been reported in other settings [6]. These dynamics suggest that professional boundaries may be reinforced not only through institutional structures but also through informal discourse, humour, and elements of the hidden curriculum [8,9].

Despite increasing advocacy for interprofessional education, qualitative research specifically exploring how medical and dental faculty and students construct their professional relationship remains limited [10]. Understanding these perceptions is particularly important in contexts such as Croatia, where medicine and dental medicine operate within the same healthcare ecosystem but are institutionally separated. Faculty perspectives are especially relevant, as attitudes embedded within the hidden curriculum may shape how future practitioners conceptualize professional roles and collaboration [9]. Although this study was conducted within one Croatian university, it addresses mechanisms of professional identity formation, hidden curriculum, and interprofessional interaction that are relevant to comparable educational and healthcare settings beyond Croatia. Insights into these dynamics may inform strategies to strengthen interprofessional collaboration, improve communication and referral practices between medical and dental professionals, support more integrated healthcare delivery, and ultimately enhance patient care [11,12].

### Aim

This study aimed to explore how Croatian medical and dental faculty members and students perceive and construct their professional relationship, and how these perceptions influence collaboration at educational and professional levels. Specifically, the study sought to examine (1) how professional identities and boundaries are articulated across groups, (2) how perceived hierarchies or stereotypes emerge within educational contexts, and (3) how structural and cultural factors shape opportunities and barriers for interprofessional collaboration.

## 2. Materials and Methods

### 2.1. Ethics

This study was approved by the Ethics Committees of the School of Dental Medicine and the School of Medicine, University of Zagreb under approval numbers 05-PA-30-XXIV-2/2021 and 641-01/21-02/01. All participants provided informed consent prior to their interviews. Before participation, all individuals received information about the study aims, interview procedures, voluntary participation, confidentiality measures, the use of audio recording, and their right to withdraw from the study at any time without consequences. To protect confidentiality, identifiers were removed from transcripts and each participant was assigned an anonymized code. Given the small academic community, care was taken to omit or generalize any potentially identifying details. Audio recordings and transcripts were stored securely on password-protected devices accessible only to the research team. In the manuscript, quotes are identified only by broad category (e.g., “Medical Student” or “Dental Professor”) to further protect participant anonymity. The study was conducted in accordance with the Declaration of Helsinki.

### 2.2. Study Design

We employed an exploratory qualitative research design to investigate interprofessional perceptions between the medical and dental professions. A qualitative approach was considered appropriate because it enabled an in-depth exploration of participants’ experiences, perceptions, and interpretations of interprofessional relationships within their educational and professional context. The study was conducted between February and August 2023 at the University of Zagreb, Croatia. Although the School of Medicine and the School of Dental Medicine are part of the University of Zagreb, they operate as separate faculties with distinct educational programmes, making this a suitable setting for exploring interprofessional perceptions within a shared academic environment. The detailed description of the study setting and participant characteristics enables readers to assess the transferability of the findings to similar educational contexts. The study is reported in accordance with the Consolidated Criteria for Reporting Qualitative Research (COREQ).

#### 2.2.1. Sampling and Participants

Participants were recruited using purposive and snowball sampling to ensure representation of key stakeholder groups: medical faculty, dental faculty, medical students, and dental students. Purposive sampling was used to recruit information-rich participants able to provide diverse perspectives on interprofessional collaboration, while snowball sampling facilitated the identification of additional eligible participants through existing professional and academic networks. One faculty participant held dual professorships in medicine and dentistry, providing interdisciplinary insight. 

Student inclusion criteria were age ≤26 years and no history of repeating an academic year. These criteria were applied to obtain a relatively homogeneous student sample and to minimize the potential influence of substantially different educational trajectories on participants’ perceptions. We aimed to include participants of both sexes and a variety of academic sub-disciplines. Some participants had family members in the other profession, which was noted as a potential factor influencing perceptions. The final sample comprised 39 participants (Table 1).

Recruitment continued until data saturation was achieved, defined as the point at which additional interviews no longer generated new codes or concepts relevant to the research questions. Saturation was assessed continuously during the analytical process through ongoing comparison of newly identified codes with existing thematic categories.

As all participants were recruited from a single university, the findings should be interpreted within this institutional context. In keeping with qualitative research methodology, the objective was not statistical generalizability but an in-depth understanding of participants’ experiences. The detailed description of the study context and participant characteristics enables readers to judge the transferability of the findings to similar educational and healthcare settings.

#### 2.2.2. Data Collection

Semi-structured, one-on-one interviews were conducted in Croatian, the participants’ native language, by two trained psychologists. The interviewers had previous experience in conducting qualitative interviews and were not affiliated with either the School of Medicine or the School of Dental Medicine, thereby reducing the potential influence of institutional hierarchy or professional relationships on participants’ responses. 

Each interview lasted approximately 40–60 min and was audio-recorded with participant consent. Interviewers also took brief field notes during and after sessions. These notes were used to document contextual observations and support data interpretation, but were not analyzed as primary data. 

A semi-structured interview guide was used, covering:

1. General perceptions of the other profession (e.g., “What is your overall view of [dentists/medical doctors] as professionals?”);
2. Experiences or observations of animosity or bias (e.g., “Have you encountered any rivalry, friction, or negative attitudes between medicine and dentistry? Can you describe what that looked like and where it comes from?”);
3. Collaboration during training (e.g., “In what ways do medical and dental students or faculty interact or collaborate during education? Can you describe any joint activities, courses, or projects—or the lack thereof?”);
4. Interprofessional collaboration in practice (e.g., “From your perspective, when and how do medical doctors and dental doctors need to collaborate in patient care?”).

The interview guide was developed specifically for this study following a review of the relevant literature and was designed to ensure consistency across interviews while allowing sufficient flexibility to explore emerging topics in greater depth.

Recordings were transcribed verbatim and reviewed against the audio for accuracy.

#### 2.2.3. Data Analysis and Reflexivity

Thematic analysis followed Braun and Clarke’s six-phase approach. Data were coded manually using Microsoft Word and Excel without dedicated qualitative data analysis software. Coding was conducted by the first author (Zora Tomić, MD), with independent verification and discussion with the psychologists who conducted interviews to enhance reflexivity. Analysis was primarily inductive, allowing themes to emerge from participants’ narratives rather than imposing a predetermined coding frame. 

The analysis comprised six iterative phases: familiarization with the data through repeated reading of transcripts; generation of initial codes; identification of potential themes; review and refinement of themes; definition and naming of themes; and preparation of the final report.

The research team reflected on their professional positions: the first author is a medical doctor with academic experience in both medicine and dentistry, while the interviewers are psychologists; this combination provided insight into both professional and psychosocial dimensions of responses.

Themes were iteratively developed through multiple readings of transcripts and discussion within the team. Although no formal inter-coder reliability statistics were calculated, analytic decisions were documented, and recurring patterns were continuously checked against the data. Saturation was assessed qualitatively, with no new themes emerging in the final interviews. Selected quotes were chosen to illustrate prominent themes.

To enhance the trustworthiness of the findings, several methodological strategies were employed. Credibility was strengthened through prolonged engagement with the data, iterative discussions among the research team, and the use of verbatim quotations to support interpretation. Dependability was supported by documenting key analytical decisions throughout the coding and theme development process. Confirmability was enhanced through regular discussions among researchers from different professional backgrounds, allowing alternative interpretations to be considered and reducing the influence of individual assumptions. Transferability was addressed by providing detailed descriptions of the study context, participant characteristics, and analytical procedures, enabling readers to assess the applicability of the findings to other settings.

## 3. Results

A total of 39 participants took part in the study. The findings presented below reflect participants’ accounts and the themes identified through thematic analysis. Their broader interpretation and implications are addressed in Section 4. Thematic analysis of the interviews revealed four overarching themes: (1) Mutual Perceptions of the Other Profession, (2) Animosity Between the Professions: Myth or Reality, (3) Collaboration at the Academic Level, and (4) Interprofessional Collaboration in Practice. Each theme includes subthemes capturing nuanced insights across medical and dental students as well as faculty members. Table 2 and Table 3 summarize the themes, subthemes, and example quotations.

### 3.1. Theme 1: Mutual Perceptions of the Other Profession

Participants consistently articulated respect toward the other profession; however, their accounts also suggested subtle processes of professional boundary construction. Interviewees frequently prefaced critical reflections with disclaimers such as “I respect them, but…”, before describing perceived differences in status, expertise, or educational scope between the professions.

Although many described medicine and dentistry as parallel healthcare professions, participants’ narratives suggested asymmetrical positioning. Medical participants often framed dentistry as a narrower domain, emphasizing medicine’s breadth and systemic scope (“we study the whole body”), whereas dental participants described experiencing implicit devaluation, particularly in educational contexts. Hierarchical distinctions were more commonly expressed through comparisons of curricular scope, perceived difficulty, and symbolic status than through open denigration.

Participants frequently described humour and “banter” among students as a socially acceptable mechanism through which perceived hierarchy was reinforced. Statements about whose studies were “harder” normalized assumptions about differential prestige while maintaining a surface of collegiality. Dental students’ accounts suggest that these micro-level interactions contributed to feelings of marginalization.

Personal exposure to the other profession was described as influencing participants’ perceptions. Students who had family members in the other profession or meaningful friendships across faculties showed greater empathy. In contrast, participants lacking such contact relied more heavily on inherited narratives from peers or faculty. Participants’ accounts suggest that professional identities were shaped not only through formal curricula but also through social proximity and interpersonal contact.

Overall, participants’ accounts indicate that mutual perceptions were characterized less by overt conflict than by an ongoing negotiation of professional status and expertise, in which symbolic hierarchy coexisted with declared mutual respect.

### 3.2. Theme 2: Animosity Between the Professions—Myth or Reality?

Participants largely rejected the existence of explicit hostility between professions. However, detailed accounts revealed recurring micro-level tensions embedded within educational and institutional structures. Rather than describing overt antagonism, participants more often referred to subtle forms of friction embedded in everyday educational and institutional practices.

Faculty frequently minimized tensions as humour or historical residue. In contrast, students—particularly from dentistry—recounted experiences of dismissive remarks during shared foundational courses. Notably, medical students were often unaware that such comments were experienced as demeaning by dental students.

Participants’ accounts indicated that interactions perceived by one profession as light-hearted competition were experienced differently by the other. These tensions were typically expressed through subtle signals regarding legitimacy and expertise.

Participants identified multiple structural sources of these tensions:

- Curricular differentiation, where differences in programme design became shorthand for competence hierarchies.
- Competitive academic cultures, particularly among high-achieving students, where comparison served to reinforce professional identity.
- Faculty influence, as senior professionals implicitly modelled attitudes toward the other field.
- Institutional politics, including competition over training domains and academic authority.

Importantly, animosity did not translate into active obstruction of collaboration. Instead, it functioned as a latent condition shaped by institutional arrangements and symbolic hierarchies. Taken together, participants’ accounts suggest that these tensions reflected professional differentiation embedded within educational and institutional contexts rather than overt interpersonal hostility.

### 3.3. Theme 3: Collaboration at the Academic Level

Academic collaboration between faculties was described as structurally minimal. Although students shared early foundational courses, participants consistently characterized these encounters as “co-presence without interaction.” Physical proximity did not translate into meaningful interprofessional engagement.

Educational structures reinforced separation beyond the preclinical years. After initial shared courses, students described complete curricular divergence, accompanied by limited awareness of the other programme’s content or competencies. Faculty similarly acknowledged restricted insight into the parallel curriculum.

This institutional separation was associated with mutual unawareness and sustained stereotypical assumptions. Participants described relying on informal narratives to understand the other profession’s role in the absence of structured collaborative learning opportunities.

Notably, collaboration within education was largely referral-oriented rather than co-management. Both groups were trained to recognize when to refer patients but not how to coordinate care. Participants’ accounts suggested that educational experiences placed greater emphasis on recognizing appropriate referrals than on developing collaborative approaches to patient management.

Participants described collaborative learning as often occurring only after graduation, if at all, with one faculty member noting that students were left to “figure it out later.”

Collectively, participants’ accounts suggest that existing educational structures provided few opportunities for meaningful interprofessional interaction during training.

### 3.4. Theme 4: Interprofessional Collaboration in Practice

In clinical contexts, participants emphasized that medical–dental collaboration becomes indispensable in specific high-complexity scenarios, including head and neck oncology, severe odontogenic infections, trauma, and systemic conditions with oral manifestations.

However, outside of acute or high-risk cases, collaboration was described as sporadic and dependent on individual initiative. Routine practice was characterized primarily by unilateral referrals without structured feedback loops, with participants frequently describing work within parallel systems rather than integrated teams.

Structural barriers reinforced this fragmentation:

- Administrative and physical separation of services;
- Lack of shared clinical records;
- Absence of routine communication channels;
- Exclusion of dental professionals from hospital-based teams;
- Time pressures discourage coordination.

These structural barriers were described as reinforcing a division of responsibility framed as “not my domain,” with collaboration largely characterized as reactive rather than routinely embedded in everyday clinical practice.

Participants’ accounts suggest that this fragmentation reflected not only individual attitudes but also organizational features of the clinical environment. Even where goodwill and mutual respect were present, structural conditions were described as limiting sustained interprofessional engagement.

Although many participants expressed a desire for stronger collaboration, they also highlighted the need for improved organizational support, communication, and coordination.

## 4. Discussion

This study examined professional relationships between medicine and dentistry in Croatia through the perspectives of faculty members and students. While mutual respect was generally expressed, participants described professional relationships as being shaped by structural separation, limited interaction, and enduring cultural perceptions. Rather than overt conflict, the findings suggest that these divisions were experienced as latent tensions maintained through educational design, interpersonal dynamics, and institutional structures.

The near-complete segregation of medical and dental curricula within the studied educational setting emerged as a central factor shaping interprofessional perceptions. Aside from a few shared basic science courses, participants described meaningful interprofessional education (IPE) as largely absent. Participants’ accounts suggested that this separation reinforced professional boundaries, perpetuated stereotypes, and limited opportunities for early collaboration. Participants expressed interest in joint workshops or projects but resisted merging curricula, indicating that while specialized training is valued, structured interaction within existing programmes is necessary to cultivate interprofessional competence. These findings align with international literature demonstrating that shared educational experiences foster mutual respect and reduce stereotypical thinking [13,14]. Similarly, the study by Hackenberg et al. showed that interprofessional exposure increases appreciation for colleagues’ expertise, even when degrees remain separate [2]. Our results further suggest that personal ties—family or close friendships across professions—mediate these perceptions, supporting intergroup contact theory and the notion that social proximity can disrupt entrenched professional stereotypes [15,16].

While participants largely denied overt rivalry, narratives revealed subtle hierarchies and implicit biases. Medical participants often framed dentistry as narrower in scope, whereas dental participants perceived devaluation of their knowledge and skills, particularly in educational settings. These asymmetrical perceptions were reinforced through humour and peer “banter,” which functioned as socially sanctioned mechanisms for maintaining status distinctions. Our findings suggest that seemingly minor expressions of rivalry may reflect broader perceptions of professional hierarchy. These subtle tensions, manifested in dismissive remarks or symbolic positioning, reflected ongoing negotiation of professional identity rather than explicit conflict. Consistent with the findings of Omisore et al., faculty influence and institutional culture may play a key role in perpetuating or mitigating these dynamics [17].

The key findings of this study can be summarized as follows: Mutual respect exists, but persistent stereotypes—such as the belief that “medics know more” or that “dentists have a narrower focus”—reflect historical hierarchies that still need to be addressed. While open animosity is rare and mostly playful, instances of disrespect, particularly toward dental students, have occurred, demonstrating counterproductive dynamics for building effective teamwork. During education, collaboration remains minimal, representing a missed opportunity to reduce mutual unawareness and stereotypical perceptions, despite a strong interest in increasing interprofessional learning experiences. In clinical practice, collaboration tends to be case-based rather than systematic, particularly in primary and preventive care, where coordination remains limited due to systemic barriers.

In summary, our findings suggest that strengthening collaboration between medicine and dentistry requires sustained efforts in both education and clinical practice. As one participant concluded, the issue is not a lack of respect, but a lack of shared practice culture—a perspective that highlights the importance of creating opportunities for collaboration throughout education and clinical practice.

### 4.1. Implications for Interprofessional Collaboration and Healthcare Practice

Our findings suggest that introducing structured interprofessional learning opportunities into medical and dental education—such as a shared “Oral-Systemic Health” course covering diabetes, periodontitis, and cardiovascular disease, along with shared prescribing responsibilities—could strengthen collaboration and support patient-centred care, consistent with recommendations by Roitman et al. and Fatahzadeh et al. [18,19]. In addition, previous studies have suggested that oral medicine-centred case conferences represent a valuable educational approach for demonstrating the relationship between oral and systemic health, promoting meaningful interprofessional collaboration, and enhancing oral health competencies among students from non-dental health professions [19]. Simulation exercises or joint case discussions, which participants themselves identified as valuable, would further support these educational approaches. These approaches are consistent with growing international evidence supporting structured interprofessional education as a means of strengthening collaborative healthcare practice.

The WHO Framework for Action on Interprofessional Education and Collaborative Practice emphasizes that shared learning provides an important foundation for collaborative practice and improved healthcare delivery, which is consistent with our findings that participants viewed early educational experiences as important in shaping interprofessional attitudes [20]. Similarly, Çınar Tanrıverdi et al. demonstrated that interprofessional education (IPE) significantly improves attitudes and professional socialization among students, further supporting the value of structured interprofessional learning opportunities [21].

Encouragingly, some participants noted that younger professionals, particularly those with greater exposure to interdisciplinary clinical settings [e.g., hospitals], showed greater comfort with interprofessional collaboration. In our study, participants involved in team settings experienced the benefits and advocated for expanding such models. This finding is supported by the study by Shwartz et al., which showed that structured interdisciplinary bedside rounds improve interprofessional communication and workplace efficiency among residents and nurses on an inpatient internal medicine unit [22]. Previous research also suggests that positive collaborative experiences can reinforce favourable attitudes towards interprofessional practice, supporting opportunities for structured collaboration during both education and clinical practice [23]. A protocol for dental clearance before certain treatments was cited by participants as a concrete way to institutionalize cooperation. This interpretation is further supported by a recent systematic review demonstrating that structured strategies integrating oral health into primary care—including interprofessional education, coordinated referral pathways, and organizational changes—can strengthen collaboration between medical and dental professionals and improve the delivery of integrated care [24]. Participants expressed optimism that greater exposure to collaborative learning and interdisciplinary practice may strengthen interprofessional relationships among future generations of healthcare professionals. Consistent with these observations and existing literature, interprofessional education could be integrated early and consistently in both medical and dental curricula, with joint modules and co-facilitated faculty engagement to foster mutual understanding. Joint student-led activities, including research projects and public health initiatives, were also highlighted as valuable opportunities to strengthen interprofessional interaction. Several participants emphasized the importance of addressing stereotypes early in training to encourage mutual respect between the professions. Together with previous evidence, our findings support further exploration of educational and organizational strategies that facilitate communication, referral practices, and integrated patient care [25,26].

Future research should evaluate the effectiveness of educational interventions and integrated models of care across different educational and healthcare settings, while also examining their impact on patient outcomes. Such evidence could guide the development of future interprofessional educational strategies and collaborative models of healthcare delivery.

### 4.2. Limitations and Strengths

This study has several limitations. First, it was conducted at a single university, and the findings should therefore be interpreted within this specific educational context. Although qualitative research does not seek statistical generalizability, readers should consider the transferability of the findings to other educational and healthcare settings in light of their own institutional contexts. Second, as with all interview-based studies, participants may have been influenced by social desirability, particularly when discussing professional relationships. Although reflexive strategies were employed throughout the analytical process, the researchers’ professional backgrounds may have influenced data interpretation. This was addressed by conducting confidential interviews with independent interviewers who were not affiliated with either faculty, thereby encouraging open discussion. Third, purposive and snowball sampling were used to recruit information-rich participants rather than to achieve statistical representativeness. In addition, the exclusion of students who had repeated an academic year may have limited the range of experiences represented within the student sample. These sampling decisions should therefore be considered when interpreting the transferability of the findings. 

Despite these limitations, the study has several strengths. Including medical and dental faculty members as well as medical and dental students provided a comprehensive perspective on interprofessional relationships across professions and stages of professional development. This diversity of participant groups strengthened the credibility of the findings by enabling comparison and corroboration of themes across different perspectives. Furthermore, it provided insight into how professional perceptions may be transmitted within educational environments and evolve over time. Finally, thematic analysis, supported by a rigorous coding process, researcher reflexivity, and collaborative review, enhanced the credibility and trustworthiness of the findings.

## 5. Conclusions

This study highlights that participants perceived the relationship between the medical and dental professions as being shaped by structural, cultural, and educational factors, despite generally expressing mutual respect. Participants described latent hierarchies, implicit biases, and limited opportunities for interprofessional engagement, particularly during education and routine clinical practice. Nevertheless, participants recognized the value of collaboration and expressed interest in structured interprofessional learning and coordinated practice. Taken together, these findings suggest that creating opportunities for structured interprofessional learning and supporting collaborative clinical practice may strengthen mutual professional understanding, facilitate interprofessional engagement, and ultimately contribute to more integrated, patient-centred care.

## Figures and Tables

**Table 1:** Study participants.

Participant Group	Total [*n*]	Female [*n*]	Male [*n*]	Academic/Study Level	Other Relevant Information
Medical Professors	8	4	4	Lecturer to Full Professor [5–30+ years teaching]	—
Dental Professors	8	6	2	Lecturer to Full Professor [5–30+ years teaching]	—
Medical Students	12	8	4	2 per year [1st–6th year]; no repeated years	All under 26; some had family in dentistry
Dental Students	12	10	2	2 per year [1st–6th year]; no repeated years	All under 26; some had family in medicine
Total	39	28	11	—	Median student age ≈ 22 years

**Table 2:** Themes on mutual perceptions and animosity, with subthemes and example quotes from participants.

Theme	Subtheme	Participant Group	Illustrative Quote
1. Mutual Perceptions of the Other Profession	Recognition of complementary roles	Dental Faculty	“We are all working towards the same goal—improving patients’ health, only with different tools.”
	Respect with underlying stereotypes	Medical Student	“Dentists are doctors, too. I don’t think they get enough credit for their knowledge of anatomy and pathology.”
	Perceived knowledge gap	Medical Student	“We study the whole body—they just do teeth.”
	Feelings of professional invalidation	Dental Student	“There’s a joke among us that med students think we’re not real doctors. It hurts when faculty reinforce that.”
2. Animosity: Myth or Reality?	Subtle hierarchies in teaching settings	Dental Student	“During anatomy class, our professor once said, ‘Now let’s see if the dental students can catch up to the med students.’ That stayed with me.”
	Minimization of bias by faculty	Medical Faculty	“There may be occasional teasing, but we have to be mindful that students internalize these hierarchies.”
	Impact of microaggressions	Dental Student	“It’s not outright hostility, but those little digs stay with you and make you feel second-rate.”
	Perception gap between students and faculty	Dental Faculty	“We don’t mean to divide them, but the structure doesn’t encourage equality.”

**Table 3:** Themes on academic and practice collaboration, with subthemes and example quotes.

Theme	Subtheme	Participant Group	Illustrative Quote
3. Academic Collaboration	Shared preclinical courses with limited interaction	Dental Student	“We sit in the same anatomy lectures, but we don’t really talk to each other—med students stay with their group, and we stay with ours.”
	Lack of structured interprofessional activities	Medical Student	“There’s no joint case study or workshop where we have to work together. I think we’d learn a lot if that existed.”
	Siloed curricula inhibit understanding	Medical Faculty	“I’m not even sure what the dental curriculum looks like after the second year. That’s part of the problem—we just don’t know what they do.”
	Missed opportunities for joint learning	Dental Faculty	“Even one shared course on patient management would help build mutual respect early.”
	Student-driven collaboration	Medical Student	“A few of us did a research project with dental students on diabetes and oral health—it opened my eyes to how interconnected our fields are.”
4. Collaboration in Clinical Practice	Necessity in complex clinical scenarios	Dental Faculty	“In head and neck oncology, we work side by side with medical doctors—there’s no way around it.”
	Fragmented coordination in routine care	Medical Faculty	“Most of the time, we refer to dentists, and that’s it. We rarely follow up or hear back—it’s not a two-way street.”
	Structural barriers in the healthcare system	Dental Faculty	“There’s no shared patient record, no standard referral system—unless we really go out of our way, collaboration doesn’t happen.”
	Mutual lack of awareness of each other’s roles	Medical Student	“I honestly don’t know what dentists are trained to manage beyond cavities and extractions.”
	Outlook for improved collaboration	Dental Student	“If we started working together more during school, we’d naturally carry that teamwork into our clinics.”

## Data Availability

The datasets supporting the conclusions of this study are not publicly available due to ethical and privacy restrictions.
